# Hygrothermal Behavior of a Washing Fines–Hemp Wall under French and Tunisian Summer Climates: Experimental and Numerical Approach

**DOI:** 10.3390/ma15031103

**Published:** 2022-01-30

**Authors:** Naima Boumediene, Florence Collet, Sylvie Prétot, Sami Elaoud

**Affiliations:** 1 Laboratoire Génie Civil et Génie Mécanique, Université de Rennes, 35704 Rennes, France; naima.boumediene@univ-rennes1.fr (N.B.); sylvie.pretot@univ-rennes1.fr (S.P.); 2 Laboratory of Applied Fluid Mechanics and Modeling, Engineering School of Sfax, Sfax 3038, Tunisia; elaoudsa@yahoo.fr

**Keywords:** heat and mass transfer, experimental study, numerical simulation, washing fines hemp wall, bi-climatic device, summer climate

## Abstract

This study experimentally and numerically investigates the hygrothermal behavior of a wall made of washing fines hemp composite under typical French and Tunisian summer climates. Actually, insulating bio-based building materials are designed in order to reduce energy and non-renewable resources consumptions. Once their multiphysical properties are characterized at material scale, it is necessary to investigate their behavior at wall scale. Washing fines hemp composite shows low thermal conductivity and high moisture buffer ability. The test wall is implemented as separating wall of a bi-climatic device, which allows simulating indoor and outdoor climates. The numerical simulations are performed with WUFI Pro 6.5 Software. The results are analyzed from the temperature, relative humidity and vapor pressure kinetics and profiles and from heat and moisture transfer and storage. The thermal conductive resistance calculated at the end of the stabilization phase is consistent with the theoretical one. The hygric resistance is consistent for simulation up to steady state. The dynamic phase under daily cyclic variation shows that for such cycles two thirds of the thickness of the wall on the exterior side are active. It also highlights sorption-desorption phenomena in the wall.

## 1. Introduction

In the context of sustainable development, the need to limit the impact of human activity on the environment has become a challenge in many sectors. In fact, the building sector is a priority area of intervention, as it represents one of the largest sources of energy consumption. According to the IEA, building construction and use accounted for about 36% of the world energy consumption in 2017, of which 30% was from the use phase [1]. To be more precise, in France, in 2019, the building sector represented 43% of the energy consumption and 14% of CO_2_ gas emissions. Heating accounted for 65% of energy consumption in the residential sector and for 43% in the tertiary sector. Cooling was low for the residential sector and accounted for 9% of energy consumption in the tertiary sector [2]. In Tunisia, the building sector accounted for 27% of the country’s total energy consumption (16% for the residential sector and 11% for the tertiary one) [3]. According to [4], since 2000, the electricity consumption peak has shifted to the middle of the day in summer and is due to the intensive use of air conditioning. Indeed, the number of air conditioners increased by 28% between 1999 and 2004 [5]. Consequently, the need for thermally efficient building envelopes with a low environmental balance is required to achieve comfortable, healthy indoor conditions.

In this context, several materials have been developed with bio-based raw materials that are used as aggregate and/or as binder. Many bio-based aggregates are investigated, such as hemp shiv, wood, barley/wheat/rape straw, corn pith and rice husks. They are used with several kinds of binders: mineral or agro-sourced (such as lime based, cement based, clay based, starch, agro-sourced from extraction) [6,7,8,9,10,11,12,13,14,15]. In general, such materials show low thermal conductivity and high moisture buffer ability. Once their multiphysical properties are characterized at material scale, it is necessary to investigate their behavior at wall scale.

The hygrothermal characterization at wall scale can be experimentally performed in a laboratory, with ambient conditions controlled on one or on both sides of the wall, or in situ. In Rahim et al. [16] and Medjelekh et al. [17], the hygrothermal behavior of walls is highlighted thanks to the monitoring of temperature and relative humidity at several depths in the walls regarding variations of ambient conditions. Several kinds of scenario are considered: isothermal conditions with step in relative humidity, simultaneous square temperature and relative humidity variations and sinusoidal temperature and RH variations. The monitoring highlights that thermal and hygric phenomena are highly coupled and show the inertia effect. Palomar et al. [18] investigated the hygrothermal performance of a brick wall under steady state winter and summer conditions. They showed that the hygrothermal stabilization of the wall was reached in the outer layers at first, and then in the inner layers. They also analyze the phenomenon of the internal condensation. Evangelisti et al. [19] investigated the thermal transmittance of three different building walls, from in situ measurements, using the UNI 10,351 standard to identify the most efficient retrofit solution. Li et al. [20] and Wu et al. [21] analyzed dynamic behavior from heat storage/release within the wall under dynamic solicitations. Chennouf et al. [22] studied a cement mortar filled with date palm fibers exposed to repeated hygric cycles. They showed a global decrease in vapor pressure of the wall during the cycles and concluded there was a drying effect during the test.

The hygrothermal characterization at wall scale can also be performed numerically. Seng et al. [23] validated their numerical heat and mass transfer model with their experimental study and then used it to highlight moisture damping and thermal insulation capacity of a hemp concrete wall. They validated that the model can be useful to compare other walls with a typical wall made of concrete. Maalouf et al. [24] performed a numerical study of a hemp concrete envelope under several French climatic conditions. The hygrothermal transfer within the walls was simulated with a 1D model. This model has been validated by comparison with experimental studies. The performance of the envelope was evaluated with the operating temperature and the interior relative humidity.

This work investigates the hygrothermal behavior of a wall made of a washing fine hemp composite [25,26]. It includes experimental and numerical approaches under static and dynamic conditions based on typical days of several French and Tunisian summer climates. This paper first presents the experimental set up and the numerical tool WUFI Pro 6.5 Software. The hygrothermal behavior of the wall is analyzed under constant temperature and vapor pressure gradients where profiles, thermal conductive resistance and hygric resistances are calculated and compared with theoretical and numerical ones. For the dynamic phase, the experimental kinetics and the temperature and vapor pressure profiles obtained at several times are given and compared with the numerical results. The temperature and vapor pressure shift and damping allow for the characterization of thermal and hygric inertia of the wall. Heat and moisture fluxes, storage and release are quantified during the last day cycle.

## 2. Methods

### 2.1. Experimental Device and Metrology

**Figure 1:**
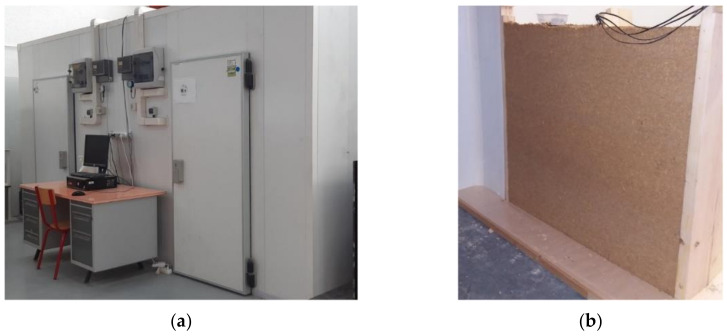
Experimental device (**a**), WFH wall (**b**).

The experimental set-up consists of a bi-climatic device made of two climatic rooms (Figure 1a). Each room is 2.35 m deep, 2.78 m wide and 2.4 m high. The floor is built of concrete; the outside walls and the ceiling are insulated with moisture and air proof polyurethane panels (U = 0.40 W/(m^2^·K)). These rooms simulate the indoor and the outdoor climate, with respective temperature and humidity ranges of 18 to 27 °C/−5 to 35 °C and of 30 to 60% RH/30 to 90% RH. For each room, the temperature is regulated by a DR4020 universal controller (Grund-Regeltechnik, Essen, Germany) that acts on convectors for heating and on cooling units for cooling. The relative humidity is regulated by a Teddington DZR-45 (Teddington, Villeneuve-la-Garenne, France) regulator that acts on a cooling unit for dehumidification and on ultrasonic humidifiers for humidification. The change of the set point is performed manually.

The test wall corresponds to a quarter of the separation wall of these rooms (Figure 1b). It is 110 cm long and 100 cm high. It consists of a 28 cm thick layer of washing fine-hemp composite (WFH), coated with a 0.3 cm thick skincoat clay plaster (SCP) on the indoor side and with a 1.2 cm thick lime-hemp render (LHR) on the outdoor side. The washing fine-hemp composite is made of a clay-based matrix with hemp shiv. The clay-based matrix is made of fines from aggregate washing mud taken from a gravels production site. The fine is stabilized with 5% of Portland cement CEM: 52.5 N CE CP2 NF from Lafarge (Paris, France) and 5% of lime-based binder (Thermo from BCB). The bio-based aggregates are CAVAC Biofibat hemp shiv (La Roche-sur-Yon, France). The hemp to binder mass ratio is 0.5 and the total water to binder mass ratio is 0.8. For the production, the hemp shiv is mixed with a part of the water by hand for about 2 min (with water to hemp mass ratio of 0.4). At the same time as washing the fine, stabilizers and water are mixed with a vertical axis concrete mixer to form the matrix paste. The mix is then poured in place and manually compacted. The mold is released after one week and the drying of the WFH is obtained after 2 months. The indoor plaster SCP is a skincoat clay plaster from CLAYTEC^®^ (Viersen, Germany), (referred as Lehm-Oberputz fein 06). The outdoor render LHR is a two layers lime-hemp render made of BSP^®^ formulated binder developed by Lhoist within the European ISOBIO project [27] and Isofin^®^ hemp. The physical properties of these composites are detailed in Section 2.4.

**Figure 2:**
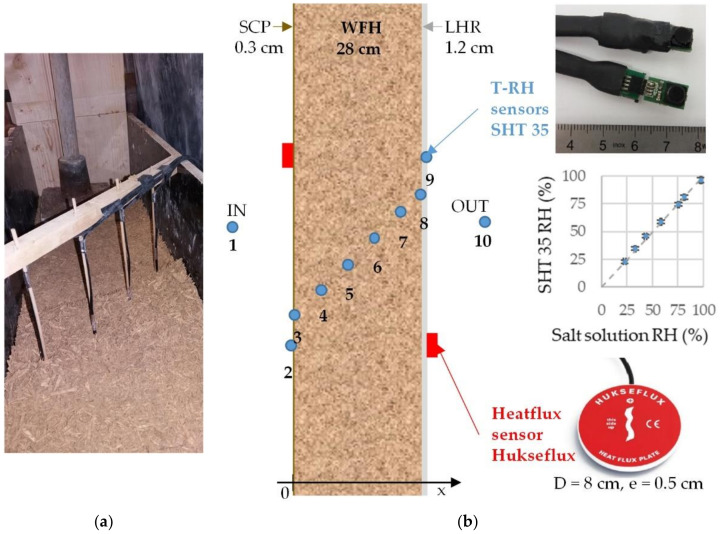
Instrumentation of the wall: (**a**) T-RH sensors implementation during the production of the wall, (**b**) sensors positions, T-RH sensors view and RH response curve, heat flux sensors.

The hygrothermal response of the wall is monitored with ten T-RH sensors (SHT35 Sensirion) located in the indoor and the outdoor ambiences, on interior and exterior exchange surfaces and distributed across the thickness of the wall (Figure 2, Table 1). Figure 2a shows the implementation of the T-RH sensors during the production of the wall. The wire of the sensors comes out of the wall vertically, to be perpendicular to heat and moisture fluxes. The positions are ensured thanks to a rigid wood mounting system. The upper part of this system is removed at the end of the production of the wall. The accuracy of the T-RH sensors is ±0.1 °C for a range from 20 °C to 60 °C of temperature and ±1.5% of relative humidity up to 80% RH at 25 °C. Prior to implementation, the calibration of the RH sensors is checked using the salt solution method (Figure 2b). Unfortunately, sensors 3 and 4 appeared to be defective at the beginning of the study. They were probably damaged during the implementation of the indoor coating (3) and by saturation (4). The heat flux is measured on the interior and the exterior surfaces of the wall with HFP01 heat flux sensors from Hukseflux (80 mm in diameter and 5 mm thick). For an operating temperature between 30 and 70 °C, the sensibility of HFP01 heat flux sensors is 50 μV/(W/m^2^). The acquisition is performed with CR1000 data acquisition system where the input range is set at ±2.5 mV, with a resolution of 0.33 μV.

### 2.2. Studied Climates

This paper considers summer climates from western and dry tropical countries. French and Tunisian cities are chosen as examples of such climates. The studied climates are suboceanic (Rennes), Mediterranean (Toulouse), arid (Kairouan) and island climate (Djerba).

**Figure 3:**
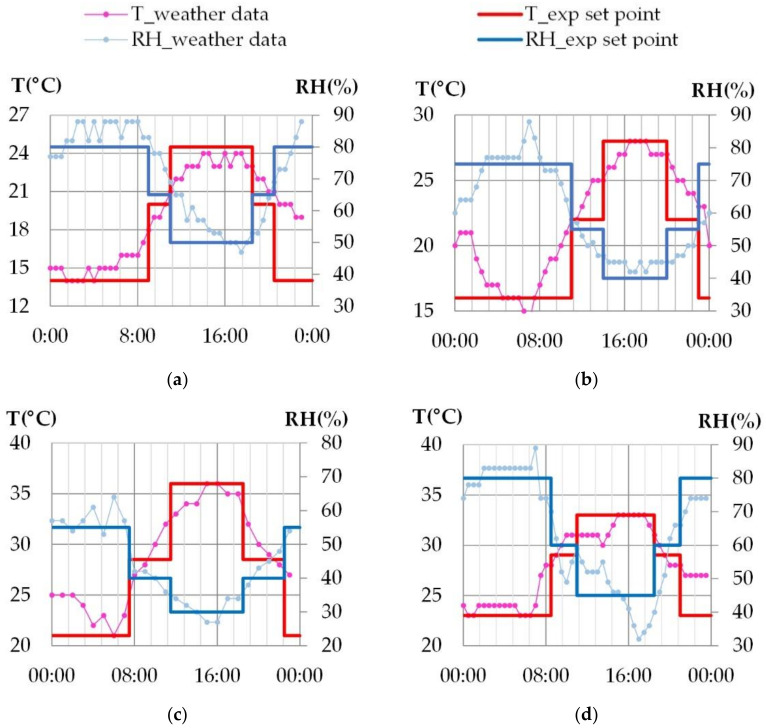
Simulated climates of French and Tunisian cities: detail of one day cycle; (**a**) Rennes, (**b**) Toulouse, (**c**) Kairouan, (**d**) Djerba.

For each climate, a typical day is chosen to be representative of the minimum and maximum 30-year average temperatures in August (France: 1981–2010 [28], Tunisia: 1961–1990 [29]) [30]. The study is performed without considering solar gains nor rain and is thus representative of north walls, cloudy days and of walls with sunscreen effect. This allows to investigate the effect of ambient temperature and relative humidity on the hygrothermal behavior of the wall. Due to the experimental device regulation, the climate cycles are simplified as shown on Figure 3, with four temperature and relative humidity steps.

**Figure 4:**
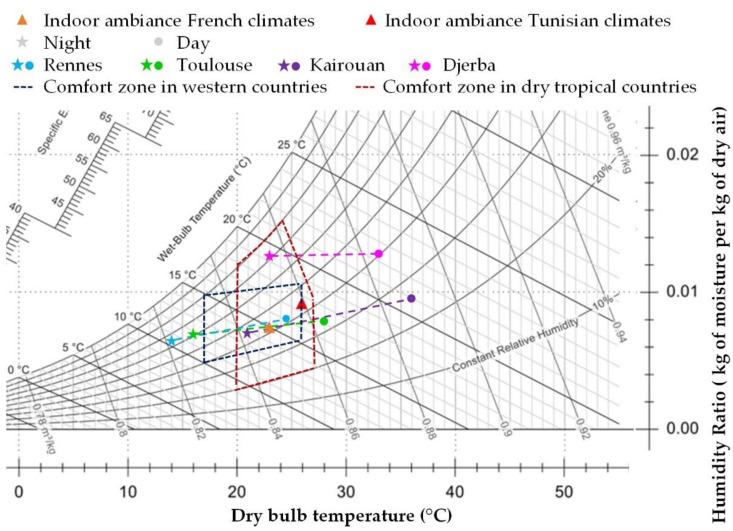
Boundary conditions: indoor and outdoor temperatures and relative humidities for the four cities.

The indoor conditions are chosen in the comfort zone to be representative of the indoor environment considered for each country in summer [31,32]: (23 °C, 50% RH) for French climates and (26 °C, 50% RH) for Tunisian climates (Figure 4).

**Figure 5:**
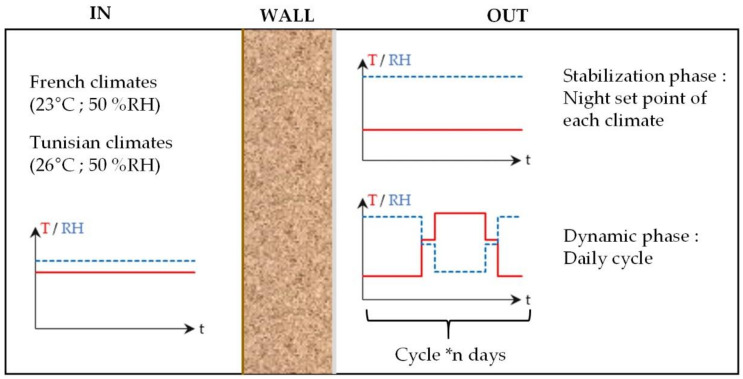
Indoor and outdoor set points during the test.

The hygrothermal study of the wall is performed in two phases. Firstly, the wall is stabilized to constant temperature and water vapor pressure gradients, corresponding to night conditions. The stabilization is considered to be reached when the relative temperature and vapor pressure variations become lower than 1%. Then, the wall is exposed to daily cyclic variations of outdoor temperature and relative humidity. The cycles are performed several times until a repeatable response of the hygrothermal behavior of the wall is obtained (Figure 5).

Figure 5 summarizes the psychrometric chart for the characteristic points for the indoor and outdoor conditions. For all climates, the temperature gradient between the indoor and the outdoor side of the wall are reversed between night and day. In France, the outdoor humidities are in the range of indoor humidity, with values higher for day than for night. In Tunisia, the outdoor humidities are in the range of indoor humidity for Kairouan. For Djerba, the outdoor humidity is much higher than the indoor one. The vapor pressure gradient is reversed between night and day for Rennes, Toulouse and Kairouan. For Djerba, it remains from outdoor to indoor in all cases.

### 2.3. Numerical Tool

In this paper, WUFI Pro 6.5 software is used to simulate the hygrothermal behavior of the wall. The phenomena taken into account in this software are conduction, storage of heat, vapor diffusion, liquid flow and moisture storage. It is based on the Kunzel model [33] where the processes relevant to heat and moisture transfer are given by two partial differential equations where temperature and relative humidity are the driving potentials.

The contact between the layers is considered as perfect.

The boundary conditions correspond to the recorded experimental ambient temperatures and relative humidities, averaged each 15 min to smooth ambient regulation variations. Thermal surface resistances are considered equal to 0.0588 m^2^·K/W on the exterior side and 0.125 m^2^·K/W on the interior side. The wall is not exposed to rain nor solar radiation.

**Figure 6:**
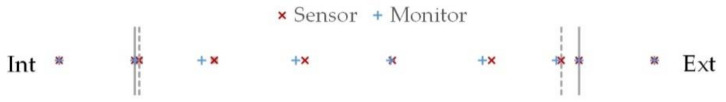
Sensors and monitors positions.

The meshing corresponds to an automatic fine grid with 100 mesh. The monitors’ positions are chosen as close as possible to the experimental sensors (Table 2, Figure 6).

### 2.4. Physical Properties of Materials

**Figure 7:**
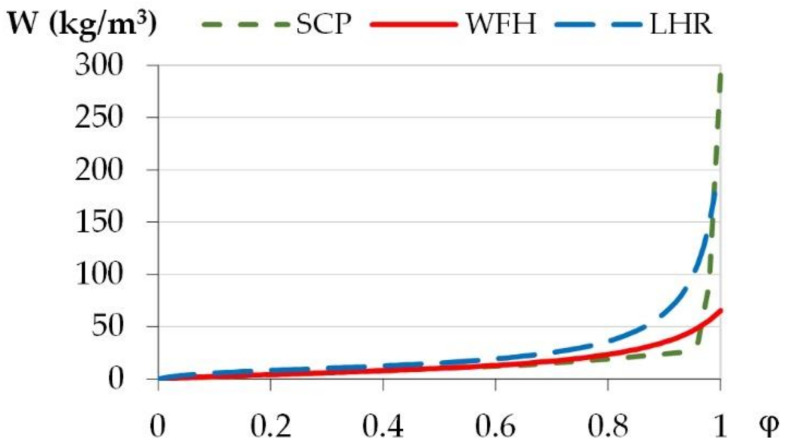
The sorption curves of materials.

Table 3 gives the physical properties of the materials. They are taken from Collet et al. [34] for the lime-hemp render (LHR), from Mazhoud [26,35] for washing fines hemp (WFH) and from WUFI Pro 6.5 database for the Skincoat Clay Plaster (SCP). For WFH and LHR, the experimental sorption curves are fitted, using least square method, with GAB model (1) [36,37,38,39]. Then, the mass water content is calculated from the GAB model each at 1% RH and the volume water content is deduced from the mass water content and from water and material densities. The sorption curves are input into Wufi material database [40] (Figure 7).

(1) $$w=w_{m}\;\frac{C1C2\varphi }{\left(1-C2\varphi \right)\left(1-C2\varphi +C1C2\varphi \right)}$$

where φ is the relative humidity (−), C1 and C2 are the fitting parameters, w_m_ is the monomolecular water content (g/g).

### 2.5. Data Analysis

#### 2.5.1. Data Analysis under Constant Temperature and Vapor Pressure Gradients

The hygrothermal behavior of the wall is analyzed from the temperature, the relative humidity and the vapor pressure evolutions. The vapor pressure *P_v_* (Pa) is calculated from experimental data of temperature *T* (°C) and relative humidity φ(−) as follows [40]:

(2) $$P_{v}=\varphi \times 610.78\;exp\left(17.08\frac{T}{234.18+T}\right)$$

Temperature, relative humidity and vapor pressure are plotted at several depths of the wall over time. Then profiles are extracted at given times (end of the stabilization phase and during the last day of the daily cyclic variation phase).

The theoretical conductive thermal resistance $R_{c}$ is calculated from the thermal resistance of each layer of the wall (3). It is equal to 2.593 (m^2^·K/W). The thermal transmittance of the wall U is calculated from the conductive thermal resistance and the surface resistances (3). The heat flux (4) and the temperature profiles are calculated from the thermal resistance and from the temperature gradient corresponding to the average values of ambient temperatures over the last 24 h of the stabilization period.

(3) U=11hi+Rc+1he; Rc=∑eiλi

(4) $$\varphi_{h}=U\left(T_{in}-T_{out}\right)$$

where U is the thermal transmittance of the wall (W/(m^2^·K)), $h_{i/e}$ are the thermal transfer coefficients at the internal/external surface (m^2^·K/W) and $R_{c}$ is the conductive thermal resistance of the wall (m^2^·K/W), $e_{i}$ is the thickness of the material *i* (m), $\lambda_{i}$ is the thermal conductivity of the material *i* (W/(m·K)), $\varphi_{h}$ is the heat flux (W/m^2^), $T_{in/out}$ are the average value of ambient indoor/outdoor temperature (°C).

The conductive thermal resistance of the wall is also obtained from the experimental and from the numerical data. The method is based on the ISO 9869 standard [41] where, according to Rasooli et al. [42] and Ficco et al. [43], the main recommendations to report an acceptable R-value are: (i) the measurement period should be multiple of 24 h and at least 72 h, (ii) the R value obtained at the end of the test does not differ by more than 5% from the value obtained 24 h before, (iii) the difference between R-value obtained from the first and the last certain number of days does not deviate more than 5%. More, the temperature gradient should be 5 K at least. To calculate the conductive thermal resistance of the wall, the surface temperatures (interior and exterior) and the heat fluxes (interior and exterior) are averaged over the last 24 h of the considered period. The conductive thermal resistance $R_{c,int}$ is calculated from the interior heat flux and the temperature gradient (respectively, $R_{c,ext}$; exterior heat flux) (5). The conductive thermal resistance of the wall is the average value of $R_{c,int}$ and $R_{c,ext}$ (6). The calculation is performed from the experimental data at the end of the stabilization phase, and from the numerical results at the end of the stabilization phase and when reaching a steady state.

(5) $$R_{c,int/ext}=\frac{\bar{T_{s,int}}-\bar{T_{s,ext}}}{\bar{\varphi_{h,int/ext}}}\;\bar{T_{s,int}}=\frac{1}{n}\sum_{j=1}^{n}T_{int,j};\;\bar{T_{s,ext}}=\frac{1}{n}\sum_{j=1}^{n}T_{ext,j};\;\bar{\varphi_{h,int/ext}}=\frac{1}{n}\sum_{j=1}^{n}\varphi_{h,int/ext,j}$$

(6) $$R_{c}=\;\left(R_{c,int}+R_{c,ext}\right)/2$$

where $R_{c,int/ext}$ are the conductive thermal resistance of the wall calculated from interior/exterior heat flux value (m^2^·K/W), $T_{s,int/ext}$ are the interior/exterior surface temperature of the wall (°C), $\varphi_{h,int/ext}$ are the heat flux measured on the interior/exterior side of the wall (W/m^2^), *n* is the number of measures over the period, $R_{c}$ is the conductive thermal resistance of the wall (m^2^·K/W).

The theoretical internal hygric resistance of the wall $R_{h}$ is calculated from the hygric resistance of each layer (7). It is equal to 7.535 × 10^9^ m^2^·s·Pa/kg. The permeance is calculated from the hygric resistance and the surface transfer coefficients. The moisture flux and the vapor pressure profiles are calculated from the hygric resistance and from the vapor pressure gradient (8). The vapor pressure gradient is calculated from the average values of ambient vapor pressures over the last 24 h of the considered period.

(7) G=11βi+Rh+1βe; Rh=∑ei×μiδair

(8) $$\varphi_{m}=G\left(Pv_{in}-Pv_{out}\right)$$

where G is the permeance of the wall (kg/(m^2^·s·Pa)), $\beta_{i/e}$ are the hygric transfer coefficient at the internal/external surface of the wall (kg/(m^2^·s·Pa)) and $R_{h}$ is the internal hygric resistance of the wall (m^2^·s·Pa/kg), $e_{i}$ is the thickness of the material *i* (m), $\mu_{i}$ is the water vapor diffusion resistance of the material *i* (−), $\delta_{air}$ is the water vapor permeability of the air (1.85 × 10^−10^ kg/(m·s·Pa)),$\;\varphi_{m}$ is the moisture flux (kg/(m^2^·s)), $Pv_{in/out}$ are the average value of ambient indoor/outdoor vapor pressure (Pa).

The internal hygric resistance of the wall is also obtained from the numerical data in the same way as for the calculation of the conductive thermal resistance. The surface vapor pressure (interior and exterior) and the moisture fluxes (interior and exterior) are averaged over the last 24 h of the considered period. The internal hygric resistance $R_{h,int}$ is calculated from the interior moisture flux and the vapor pressure gradient (respectively, $R_{h,ext}$; exterior moisture flux) (9). The internal hygric resistance of the wall is the average value of $R_{h,int}$ and $R_{h,ext}$ (10). The calculation is performed from the numerical results at the end of the stabilization phase and when reaching a steady state.

(9) $$R_{h,int/ext}=\frac{\bar{Pv_{s,int}}-\bar{Pv_{s,ext}}}{\bar{\varphi_{m,int/ext}}}\;\bar{Pv_{s,int}}=\frac{1}{n}\sum_{j=1}^{n}Pv_{int,j};\;\bar{Pv_{s,ext}}=\frac{1}{n}\sum_{j=1}^{n}Pv_{ext,j};\;\bar{\varphi_{m,int/ext}}=\frac{1}{n}\sum_{j=1}^{n}\varphi_{m,int/ext,j}$$

(10) $$R_{h}=\;\left(R_{h,int}+R_{h,ext}\right)/2$$

where $R_{h,int/ext}$ are the hygric resistance of the wall calculated from interior/exterior moisture flux value (m^2^·s·Pa/kg), $Pv_{s,int/ext}$ are the interior/exterior surface vapor pressure of the wall (Pa), $\varphi_{m,int/ext}$ are the moisture flux measured on the interior/exterior side of the wall (kg/(m^2^·s)), n is the number of measures over the period, $R_{h}$ is the hygric resistance of the wall (m^2^·s·Pa/kg).

#### 2.5.2. Data Analysis under Daily Cyclic Variation

**Figure 8:**
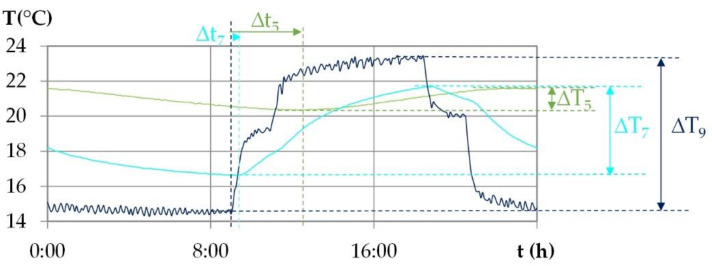
Example of identification of the temperature shift phase Δt between the surface (9) and given points (5 and 7) and of the temperature amplitude at the surface and at given points.

During the dynamic solicitation under daily cyclic variation, the heat and moisture transfer are studied regarding the shift phase and the damping of the temperature and of the vapor pressure over the thickness of the wall. The shift phase Δt is the time that separates the excitation and the response to the excitation. The damping factor is the ratio between the amplitude at the surface and the amplitude at the given point. Figure 8 shows, for example, the temperature shift phase for position 5 and 7. The temperature damping factor at these positions are equal to $\Delta T_{5}/\Delta T_{9}$ and $\Delta T_{7}/\Delta T_{9}$.

Then, the heat and moisture flux variation versus time are studied at the interior and exterior surfaces of the wall. Finally, the heat storage/release are calculated on the interior and exterior surfaces of the wall by integrating the incoming/outgoing heat fluxes over time. The total heat storage/release are the sum of the values on both surfaces (11). In the same way, the moisture storage and release are calculated on each surface and the total is the sum of the values on both surfaces (12).

(11) $$Q_{h,int/ext}=\int_{t}^{}\varphi_{h,int/ext}.dt;\;Q_{h}=Q_{h,int}+Q_{h,ext}$$

(12) $$Q_{m,int/ext}=\int_{t}^{}\varphi_{m,int/ext}.dt;\;Q_{m}=Q_{m,int}+Q_{m,ext}$$

where $Q_{h,int/ext}$ are the heat at the interior/exterior surface of the wall (W/m^2^), $\varphi_{h,int/ext}$ are the heat flux on the interior/exterior surface of the wall (W/m^2^), t is the time (s), $Q_{h}$ is the total heat (W/m^2^), $Q_{m,int/ext}$ are the moisture at the interior/exterior surface of the wall (kg/m^2^), $\varphi_{m,int/ext}$ are the moisture flux on the interior/exterior surface of the wall (kg/(m^2^·s)), $Q_{m}$ is the total moisture (kg/m^2^).

## 3. Results and Discussion

### 3.1. Stabilization Phase: Constant Temperature and Vapor Pressure Gradients

The first phase under constant temperature and water vapor pressure gradients aimed to stabilize the wall before cyclic solicitation. It lasted between 7 and 17 days (7 for Rennes, 15 for Djerba and Kairouan, 17 for Toulouse). This allowed the relative temperature and vapor pressure variations to become lower than 1% for Rennes and 0.5% for the other cities, during the last 24 h, over the entire thickness of the wall.

#### 3.1.1. Profiles

During the stabilization phase at night conditions, the indoor temperature is higher than the outdoor one for all cities. This induces a temperature gradient, and thus a heat flux, from the indoor side to the outdoor side. The indoor vapor pressure is higher than the outdoor ones for Rennes, Toulouse and Kairouan. This induces a vapor pressure gradient, and thus a moisture flux, from the indoor side to the outdoor side. For Djerba, the vapor pressure is higher on the indoor side than on the outdoor side. This induces a moisture flux opposite to heat flux. Under such conditions, the heat transfer is quickly established (within about 4 to 5 days). Two cities are chosen to analyze the temperature profiles: Kairouan is an example for cities with parallel heat and moisture fluxes and Djerba, with opposite heat and moisture fluxes. All cities are included for water vapor pressure profiles.

**Figure 9:**
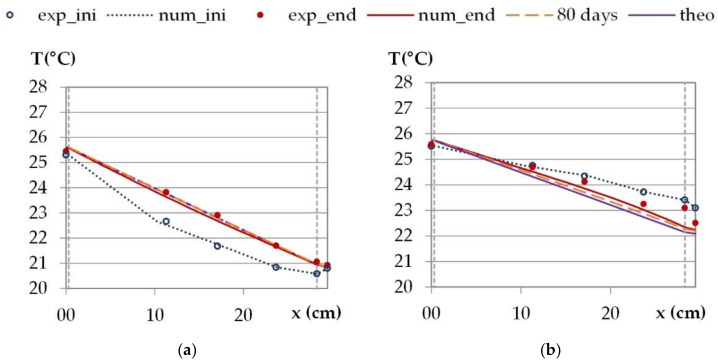
Temperature profiles under constant temperature and vapor pressure gradients, theoretical, experimental and numerical average values over the last 24 h of the stabilization phase: (**a**) climate of Kairouan and (**b**) climate of Djerba (right).

**Figure 10:**
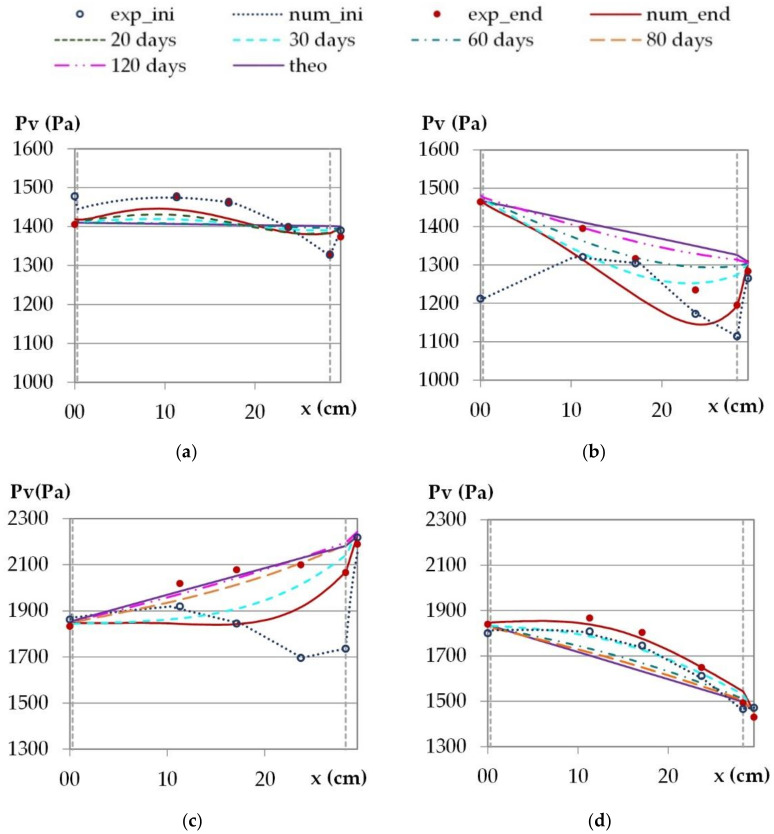
Vapor pressure profiles under constant temperature and vapor pressure gradients, theoretical, experimental and numerical average values over the last 24 h of the stabilization phase: (**a**) climate of Toulouse, (**b**) climate of Rennes, (**c**) climate of Djerba, (**d**) climate of Kairouan.

Figure 9 and Figure 10 give the temperature and vapor pressure profiles over the wall. They correspond to average values over the last 24 h of the phase under constant temperature and vapor pressure gradients. The theoretical profiles are linear as they are calculated, under constant temperature gradients, from heat flux and thermal resistances.

Regarding the temperature profiles, for cities with parallel heat and moisture fluxes (Kairouan as an example), the experimental and numerical profiles at the end of the stabilization phase show very high agreement with the theoretical ones. This shows that a steady state is nearly reached from a thermal point of view and allows us to confirm the thermal conductivity values and the sensors positions. The relative discrepancy between the theoretical profile at steady state and the numerical profile is lower than 1% all over the wall thickness after 20 days of simulation for all cities. For Djerba, the experimental and numerical profiles at the end of the stabilization phase differ from the theoretical one at a steady state. Besides, there is also a discrepancy between the numerical and the experimental profiles. The main differences are observed on the outdoor side of the wall where the experimental data are higher. These discrepancies between profiles (experimental, numerical and theoretical) are probably due to hygric phenomena with sorption–desorption effect that induce heat transfer and opposite heat and moisture fluxes.

For all the cities, the simulations are performed for much higher durations. After 80 days, a steady state is nearly reached in all cases. After 180 days, the thermal steady state is fully reached in all cases, and the thermal resistance of the wall is calculated.

Regarding the water vapor profiles (Figure 10), for the climate of Toulouse, there is nearly no water vapor pressure gradient, so the vapor pressure is becoming constant across the wall thickness. The experimental and the numerical results are close to the theoretical ones and the profile is almost established over all the thickness of the wall at the end of the stabilization phase. For the other cities, there are more discrepancies than for the temperature profiles between the theoretical profile at a steady state, the experimental and the numerical results at the end of the stabilization phase. A steady state is not fully reached at the hygric point of view. At the end of the stabilization phase, the numerical profiles are delayed compared to the experimental ones. This may be due to underestimation of the vapor diffusion, which is calculated from the vapor permeability and the main adsorption curve. In order to identify the time needed to reach the hygric steady state, the simulation of the stabilization phase is performed over a much longer stabilization time. A steady state is considered to be reached when there is less than 2% of relative discrepancy over the entire wall thickness. The time needed to reach a steady state depends on the hygric solicitation and is thus different for the four cities.

For the climate of Rennes, the theoretical water vapor pressure decreases across the thickness of the wall from inside to outside. The experimental results are getting close to the theoretical ones over the first half of the thickness of the wall, on the interior side. The numerical results show similar trends as the experimental ones. At the end of the phase of stabilization, a steady state is partially reached for experimental results. According to the simulation, a steady state would be reached after 120 days.

For the climate of Djerba, the theoretical water vapor pressure increases across the thickness of the wall from inside to outside. The experimental results are become closer to the theoretical ones when the interior side is over three quarters of the thickness. The numerical results show discrepancies with the theoretical and the experimental results. This higher discrepancy is maybe due to the fact that heat and moisture fluxes are opposite. According to the simulation, the time needed to reach a steady state is 80 days, even if the experimental results show that the vapor pressure profile is nearly established.

For the climate of Kairouan, the numerical results show the same trend as the experimental ones. They show an increase in vapor pressure over the thickness of the wall, compared to the initial profile, even if at a steady state the vapor pressure will become lower. This highlights desorption phenomena induced by heat transfer. According to the simulation, the time needed to reach a steady state would be 60 days.

Finally, the numerical results show the same trends as the experimental ones when heat and moisture fluxes are in the same direction. When heat and moisture fluxes are opposite, there is more discrepancy. The time needed to reach the hygric steady state differs from one climate to another. It is linked to the hygric inertia of the wall and depends on its initial and steady states.

#### 3.1.2. Heat and Moisture Fluxes, Thermal and Hygric Resistances

Regarding heat transfer and thermal resistances, Table 4 gives the experimental results at the end of the stabilization phase. Table 5 and Table 6 give the numerical results at the end of the stabilization phase and after 180 days, respectively. These tables give the interior and exterior surface temperatures, the interior and exterior heat fluxes and the R_c_ values (interior, exterior, average).

At the end of the stabilization phase, the experimental heat fluxes are close to the numerical ones on the interior side and on the exterior side for Rennes, Toulouse and Kairouan. Both for the experimental and the numerical results, the heat fluxes are the most important with the highest temperature gradient. There is discrepancy between the interior and the exterior heat fluxes for all cities. This is probably due to thermal inertia effect. After 180 days, the numerical heat fluxes are the same on the interior and the exterior side of the wall. This underlines that a steady state is reached for all the cities.

At the end of the stabilization phase, for the four cities, the experimental R_c_ values are consistent with the theoretical ones, with relative errors lower than 5%. Even if the temperature gradient is lower than 5K in the case of the climates of Kairouan and of Djerba, the experimental R_c_ values are consistent with the theoretical ones as the measurement is performed at the end of the stabilization under constant temperature and vapor pressure gradient. The numerical results are close to the experimental ones (discrepancies lower than 6%), except for Djerba where the discrepancy reaches 22.5%. In this case, the heat and the moisture fluxes are opposite. This configuration is more difficult to simulate. The numerical heat fluxes overestimate the experimental ones and induce lower thermal resistance values.

For longer simulation duration, for each city, the numerical R_c_ values become constant as a steady state is reached. At 180 days, the numerical R_c_ values are the same for all the cities and in accordance with the theoretical ones (discrepancy lower than 0.6%).

Regarding moisture transfer and hygric resistances, Table 7 and Table 8 give the numerical results at the end of the stabilization phase and after 300 days, respectively.

At the end of the stabilization phase, the moisture fluxes are impacted by the initial hygric state of the wall. For the climate of Djerba and of Kairouan, the moisture flux is nearly zero on the interior surface, as the numerical water vapor pressure profile is horizontal near this surface. On the exterior surface, the moisture flux is in line with the vapor pressure gradient. For the climate of Toulouse, the vapor pressure gradient is very low and the moisture flux is negligeable but not established. For the climate of Rennes, the moisture flux is important on both surfaces of the wall. It is in line with the vapor pressure gradient on the interior side of the wall and opposite on the exterior side. This highlights that the moisture fluxes are not established in the wall. The hygric resistances are not calculated as they would not be consistent in these conditions.

After 300 days, for all the cities, the moisture fluxes on the interior and on the exterior side of the wall are the same (maximal deviation of 6% for Rennes). A steady state is very close to being reached and the numerical R_h_ values become close between the four climates (deviation lower than 5%). These numerical R_h_ values are consistent with the theoretical ones with relative errors lower than 13%.

It should be underlined that the time needed to reach a steady state is much higher for hygric phenomena than for thermal ones.

### 3.2. Dynamic Solicitations: Daily Cyclic Variations of Outdoor Conditions

#### 3.2.1. Kinetics Results

**Figure 11:**
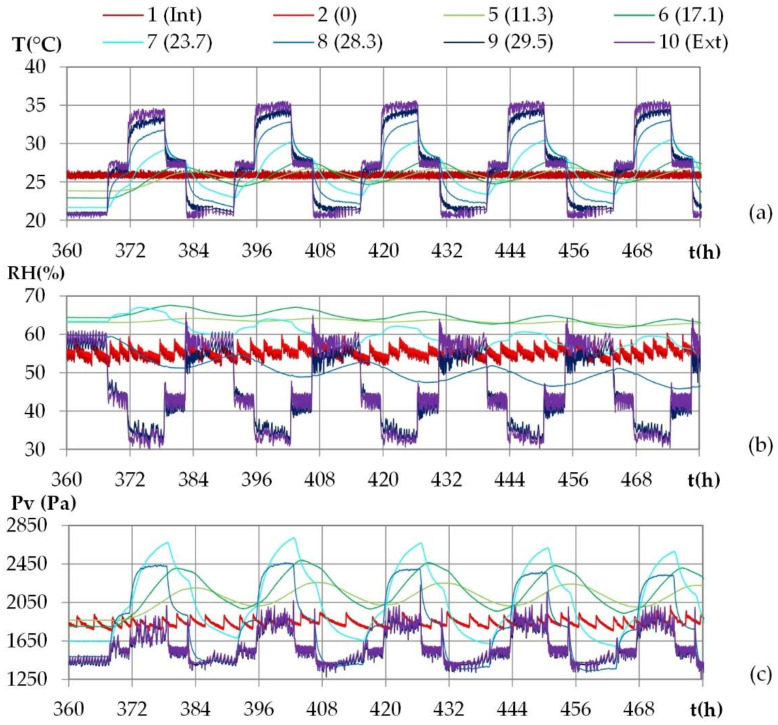
Experimental kinetics of: (**a**) temperature, (**b**) relative humidity and (**c**) vapor pressure during the dynamic cycles at several depths in the wall, for the climate of Kairouan.

For all the cities, the results show the same trends. Figure 11 gives the experimental results for the climate of Kairouan as an example. It shows the kinetics of temperature, of relative humidity and of water vapor pressure at several positions: ambient conditions, exchange surfaces and different depths in the wall.

For all the cities, the ambient indoor and outdoor conditions are in good agreement with the set points. There are low fluctuations induced by the regulation, as indicated previously they are smoothed for the simulation. The vapor pressure results from the temperature and from the relative humidity. At the set point change, the relative humidity fluctuation is magnified by the temperature variation, resulting in a peak in vapor pressure.

During the first cycles, the temperature beams increase, and the relative humidity beams decrease all over the wall. Actually, the wall was stabilized during night conditions, and thus at the lowest temperatures and at the highest relative humidities. Regarding the water vapor pressure, at the beginning of the dynamic phase, the water vapor pressure is related to the water content at the end of the stabilization phase. The water vapor progressively decreases all over the wall as the cycles are repeated. This feature highlights a progressive drying of the wall during the test.

For all the cities, the cycles repeat from the third cycle. At each position, the curves of the fourth and the fifth cycles are superimposed, with relative deviations lower than 1% in temperature and in vapor pressure for Rennes, Toulouse and Djerba (2% for Kairouan). The analysis of the hygrothermal behavior of the wall is therefore performed on temperature and water vapor pressure from the last cycle, for each city.

**Figure 12:**
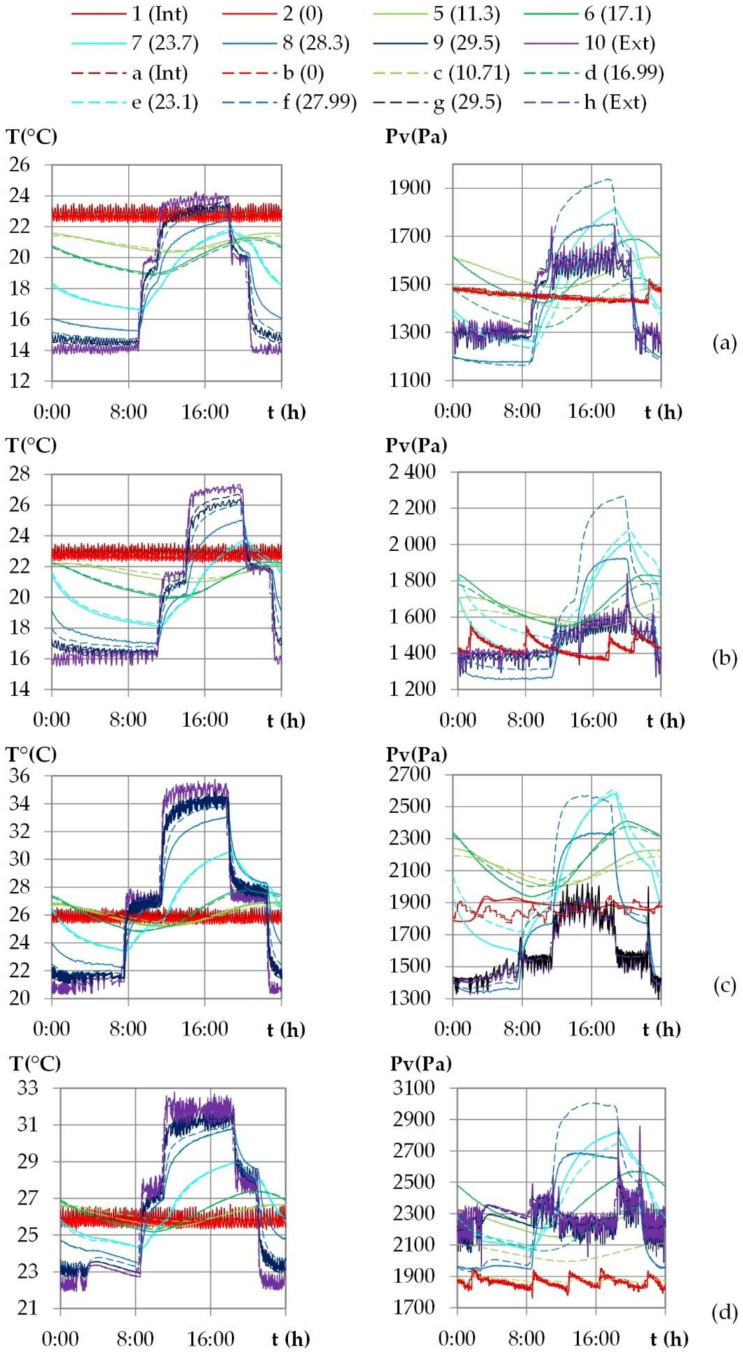
Variation of temperature and vapor pressure within the wall during the last stabilized cycle for the climate of: (**a**) Rennes, (**b**) Toulouse, (**c**) Kairouan, (**d**) Djerba. Experimental results: continuous lines; numerical results: dotted lines.

The experimental results given in Figure 12 show that for both temperature and water vapor pressure, the signals at the exterior surface and close to it show the same shape as the ambient signal. When moving from outdoor to indoor, the signal evolves gradually towards a sinusoid, between x = 23.7 cm and x = 17.1 cm, and the signal shift increases.

Figure 12 superimposes the numerical kinetics of temperature and of vapor pressure (dotted lines) at different positions to the experimental ones. The numerical ambient signal, obtained by smoothing the experimental one, is representative of ambient conditions and does not induce delay at the set point change. The time step of 15 min chosen for the smoothing is consistent to reduce regulation fluctuation without impacting the response of the wall.

The numerical kinetics of temperature are consistent with the experimental results, for all the cities and at all positions, except between the lime-hemp render and the washing fine–hemp composite. The numerical curve evolves too rapidly with a too high an amplitude. This may be due the thermal diffusivity used for the numerical simulations as the thermal conductivity is validated with the stabilization phase study.

For Rennes, Toulouse and Kairouan, the numerical kinetics of water vapor pressure are consistent with the experimental results, except between the lime-hemp render and the washing fine–hemp composite. This may be due to the deviation in temperature and to the hygric diffusivity used for the simulations. For Djerba, there is more discrepancy, with consistent numerical amplitude but with underestimation of vapor pressure value in the core of the wall. In addition to the factors identified for the other cities, this may also be due to the fact that heat and moisture fluxes are in the same direction at day and opposite at night.

**Figure 13:**
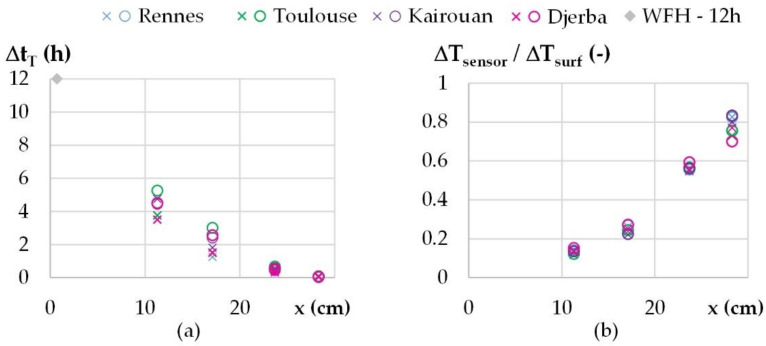
Dynamic thermal parameters versus location for all the cities (×/o: calculated during increasing/decreasing temperature step): (**a**) Shift of temperature and theoretical value for a WFH layer of 28.8 cm, (**b**) damping factor.

For temperature, Figure 13a gives the variation of the shift versus the position. The results from the different cities are close to each other, as expected, as it depends on the hygrothermal properties of the wall. At mid-thickness, the shift is about 3 h. The variation is consistent with the theoretical value: following the relation given in [44], with the thermophysical properties of WFH, a phase shift of 12 h is obtained for a WFH layer of 28.8 cm. The amplitudes are damped more and more when moving from the outdoor side to the indoor side of the wall, as shown on Figure 13b. As for the shift, the results from the different cities are similar. At mid-thickness of the wall, the damping factor is about 0.2. For the water vapor pressure, higher amplitudes than the ambient ones are observed in the wall: near the outdoor side of the wall for all the cities, and all over the wall for Toulouse and Kairouan. This leads to water vapor pressure values higher than the maximum ambient values and lower than the minimum ambient ones. This highlights sorption–desorption phenomena in the wall, magnified by temperature variation.

**Figure 14:**
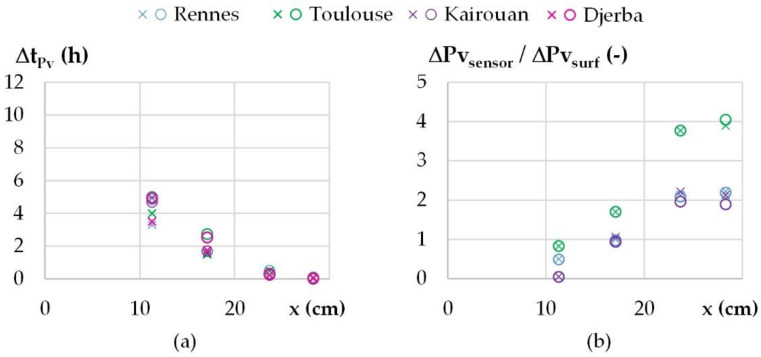
Dynamic hygric parameters versus location for all the cities (×/o: calculated during increasing/decreasing vapor pressure step): (**a**) Shift of vapor pressure, (**b**) damping factor.

For water vapor pressure, the shifts over the wall are similar to those obtained for temperature in Figure 14a. The damping factor is not calculated for Djerba because the ambient vapor pressure variation is too low. For the other cities, the damping factors reach values higher than 1, showing that the vapor pressure in the wall is not only induced by the ambient value but also by sorption–desorption effects in the wall. Similar results are obtained for Rennes and Kairouan, and higher values are obtained for Toulouse.

#### 3.2.2. Profiles over Time

**Figure 15:**
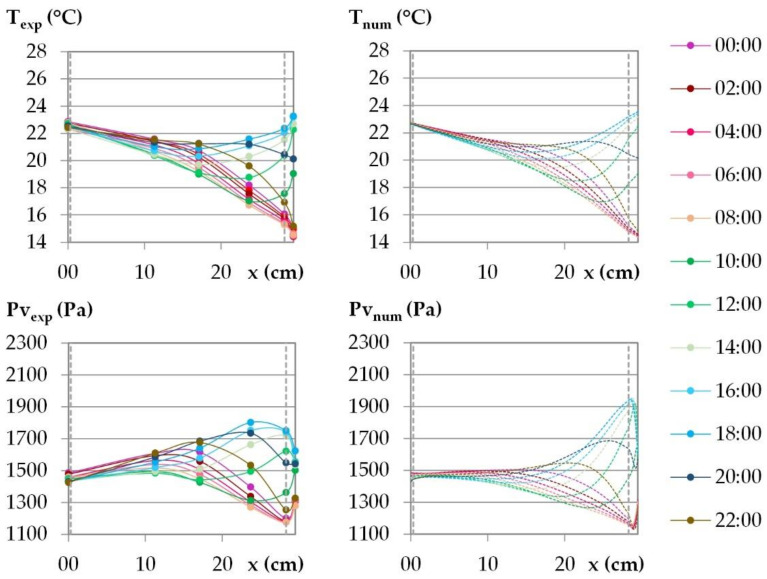
Temperature and vapor pressure profiles during the last day of the dynamic solicitation phase for the climate of Rennes. Points: experimental values; dotted lines: numerical values.

**Figure 16:**
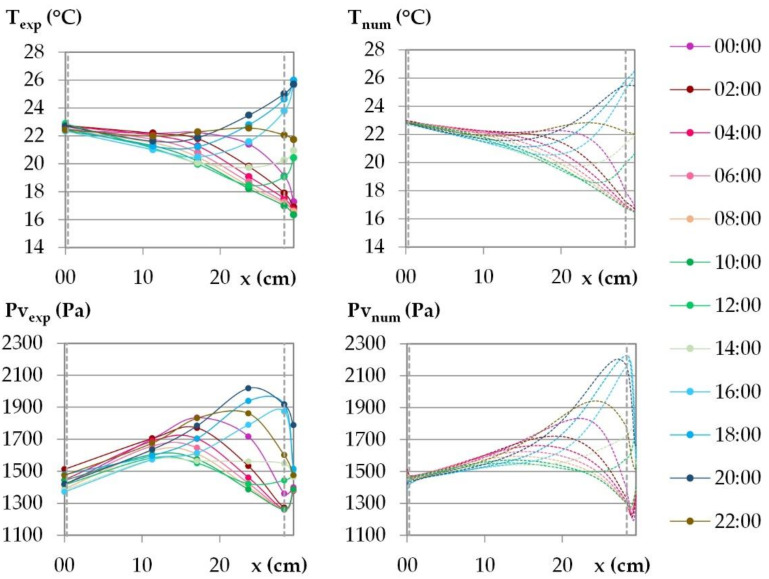
Temperature and vapor pressure profiles during the last day of the dynamic solicitation phase for the climate of Toulouse. Points: experimental values; dotted lines: numerical values.

**Figure 17:**
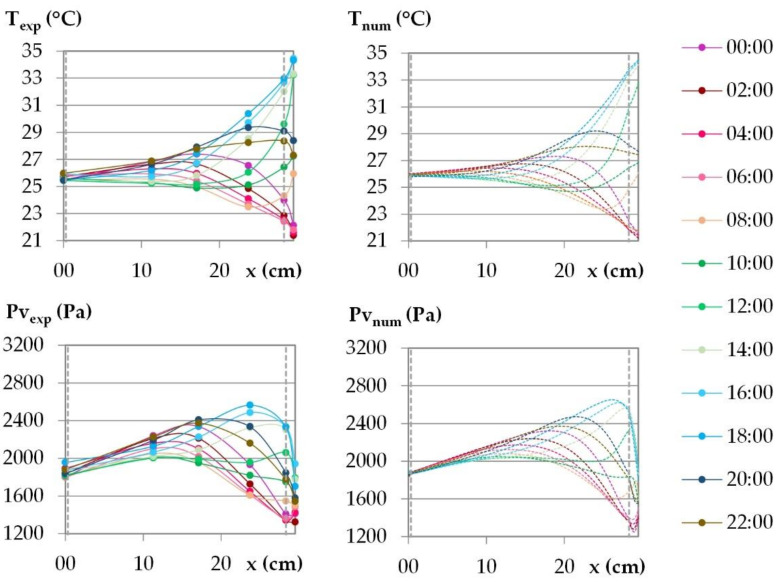
Temperature and vapor pressure profiles during the last day of the dynamic solicitation phase for the climate of Kairouan. Points: experimental values; dotted lines: numerical values.

**Figure 18:**
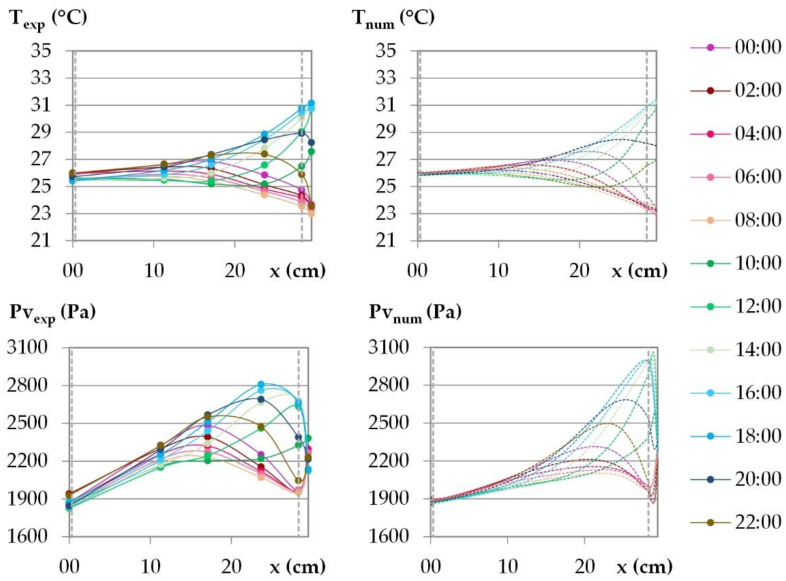
Temperature and vapor pressure profiles during the last day of the dynamic solicitation phase for the climate of Djerba. Points: experimental values; dotted lines: numerical values.

The temperature and the water vapor profiles are plotted at several times during the last day of the dynamic study in Figure 15 and Figure 16 for the French cities and in Figure 17 and Figure 18 for the Tunisian cities.

During the day, for all the cities, on the interior surface of the wall, the temperature and the vapor pressure are quasi-constant while on the outdoor surface they evolve in relation to the set points.

For temperature, regarding the experimental results, the temperature beams show low amplitude over the first third of the wall thickness on the interior side. For the French cities, the temperature gradient is negative from the indoor to the outdoor side of the wall. There are no heat gains through the interior surface of the wall when the solar gains are not considered. For the Tunisian cities, the temperature gradient on the first third of the thickness of the wall are slight and reverse during the day, with a negative gradient from the indoor side to the outdoor side during the day, and the reverse at night. This induces slight gains at night and losses during the day through the interior surface of the wall. On the exterior side of the wall, the temperature decrease at night is more pronounced for French cities due to chilly nights, the temperature increase during the day is higher for the Tunisian cities as the climate is hotter. The temperature gradients on the exterior surface of the wall evolve noticeably in amplitude and direction during the day, following the outdoor ambience solicitation. The heat transfer on the exterior side leads to a storage/release with a rapid increase/decrease in the temperature in the exterior layers and then delayed over the thickness of the wall. Finally, the two thirds of the wall thickness on the exterior side are active, and contribute to limiting, or even avoiding, heat gains on the interior side. Regarding the numerical results, the profiles are highly consistent with the experimental ones. As the solicitation signals are smoothed for input to the numerical simulation, the numerical amplitude of the temperature beam is slightly lower than the experimental one on the interior side of the wall. On the two thirds of the wall thickness on the exterior side, the numerical temperature beam meets the experimental one.

For vapor pressure, regarding the experimental results, the vapor pressure profile shows a low amplitude–increasing beam throughout the cycle on the interior side of the wall at a third of the wall thickness. This induces a slight flux from the wall to the indoor ambience. It is observed for all the cities, with greater value for Tunisian cities than for French ones. On the exterior side of the wall, a higher vapor pressure amplitude is observed, connected with the ambient solicitations. The vapor pressure gradient close to the exterior side of the wall reverses during the daily cycle. At the end of the night, before the set-point change, a minimum value of vapor pressure is observed between the LHR and WFH layer and a maximum one at mid-thickness. The moisture flux occurs both from the inner part of the wall and from the outdoor side. Throughout the day, the vapor pressure increases on the exterior part of the wall and the lower value moves to the inner part of the wall. Then, the vapor pressure goes on increasing in the outdoor part of the wall. It leads to a peak between the LHR and the WFH, which moves to the inner part of the wall as the day goes on. As underlined when studying kinetics, the vapor pressure reaches values higher than the ambient ones. They are induced by the temperature increase, and highlight desorption phenomena which cause moisture flux in the wall and through the exterior surface. The smoothing of the ambient temperature and relative humidity signals induce numerical vapor pressure variations that are slightly lower than the experimental ones on the surfaces, which is especially noticeable on the interior side. For all the cities, the numerical profiles over the wall have the same trend as the experimental ones. However, the high/low vapor pressure peaks induced by desorption/sorption phenomena are thinner and closer to the exterior surface than the experimental ones. They induce a higher vapor pressure gradient towards the desorption/sorption point. The over and underestimation of vapor pressures may be explained by the fact that WUFI Pro does not take into account the hysteresis phenomena. The numerical amplitude of vapor pressure propagates less in the thickness of the wall than the experimental amplitude. As observed for the stabilization phase, the vapor diffusion seems underestimated. Finally, the vapor pressure amplitude is overestimated in the third part of the thickness of the wall on the exterior side while it is underestimated elsewhere.

#### 3.2.3. Heat and Moisture Fluxes during Daily Cyclic Variations

**Figure 19:**
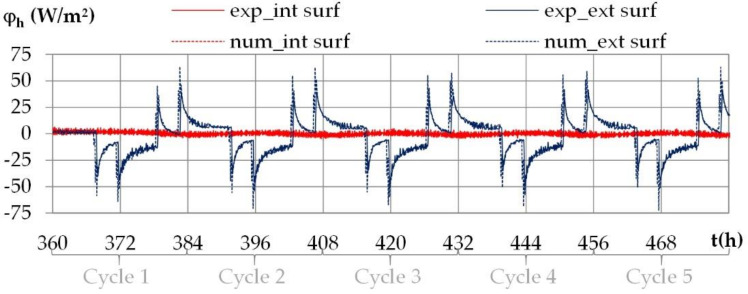
Experimental and numerical heat fluxes on the interior and exterior surfaces during the daily cycles for the climate of Kairouan.

The variation of heat fluxes on the interior and on the exterior surfaces of the wall under daily cyclic variations shows the same trends for all the cities. Figure 19 gives as an example of the experimental and numerical heat fluxes with the climate of Kairouan.

The heat flux is positive when heat flows from the indoor side to the outdoor side. Like for the temperature and the vapor pressure, the heat flux repeats during the cycles. On the interior surface, the heat flux is very small. On the exterior surface, the heat flux is induced by the outdoor temperature variation, with heat flux peak at the set point change, up to 30 W/m^2^ for Rennes, Toulouse and Djerba and 40 W/m^2^ for Kairouan. At the end of the second step in the daily cycle, the heat flux is about −5 W/m^2^ for Rennes, −9 W/m^2^ for Toulouse, −10 W/m^2^ for Djerba and −12 W/m^2^ for Kairouan, in line with the temperature gradients.

**Figure 20:**
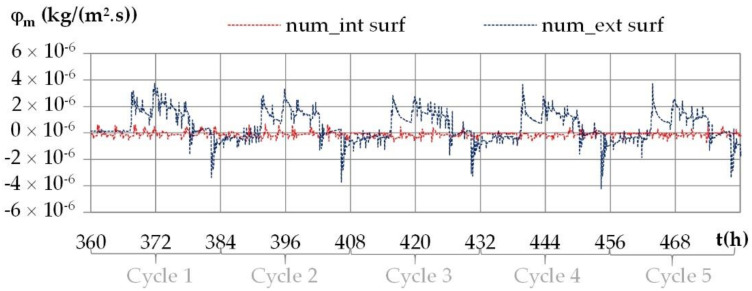
Numerical moisture fluxes on the interior and exterior surfaces during the daily cycles for the climate of Kairouan.

The variation of moisture fluxes on the interior and on the exterior surfaces of the wall under daily cyclic variations shows the same trends for all the cities. Figure 20 gives an example of the numerical moisture fluxes with the climate of Kairouan.

**Figure 21:**
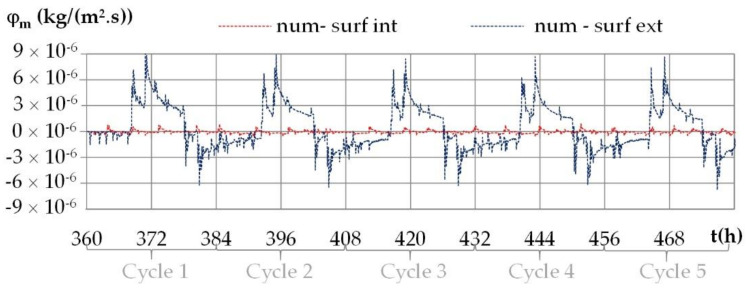
Numerical moisture fluxes on the interior and exterior surfaces during the daily cycles for the climate of Djerba.

The moisture flux is positive when moisture flows from the indoor side to the outdoor side. Like previously, the moisture flux repeats during the cycles. The moisture flux exhibits higher fluctuations than the heat fluxes as the ambient vapor pressure fluctuate more than the ambient temperature. On the interior surface, the moisture flux is close to zero (see in the Figure 21). On the exterior surface, the moisture flux shows a peak at the set point change, with intensity up to 3 × 10^−6^ kg/(m^2^·s) for Kairouan, 4 × 10^−6^ kg/(m^2^·s) for Rennes, 5 × 10^−6^ kg/(m^2^·s) for Toulouse and 7 × 10^−6^ kg/(m^2^·s) for Djerba. For Djerba, like for the three other cities, the moisture flux reverses during a daily cycle even if the ambient vapor pressure gradient remains from the outdoor side to the indoor side. The incoming/outgoing moisture fluxes are thus not only due to the ambient vapor pressure gradient but also to sorption–desorption effects enhanced by the temperature variation over the wall.

#### 3.2.4. Heat and Moisture Storage and Release during Daily Cyclic Variations

**Figure 22:**
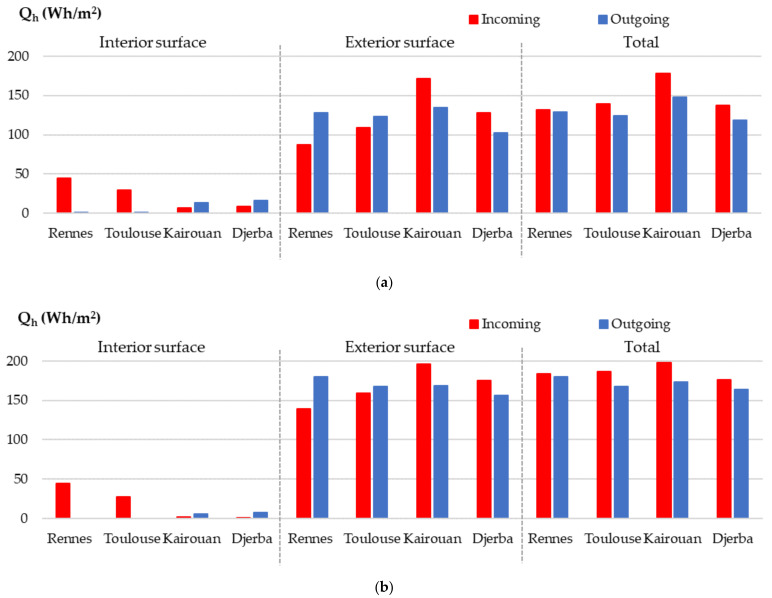
Incoming and outgoing heat during the last cycle on each surface of the wall and total of both surfaces. (**a**) Experimental results on heat; (**b**) numerical results on heat.

Figure 22 gives the incoming and outgoing heat calculated by integrating experimental and numerical fluxes versus time during the last cycle on each surface of the wall and the total of both surfaces.

Regarding the experimental results, for all the cities, the incoming and outgoing heats on the interior surface are light, linking the temperature profile with a low temperature gradient over the first ten centimeters. On the exterior surface, the incoming and outgoing heat is higher than on the interior surface, as it is exposed to a summer climate. However, the heat flux coming from the outdoor side of the wall does not reach the indoor side. The heat stored during the day is partially or completely released at night on the exterior side.

For the climate of Rennes, the incoming heat on the interior surface is half the incoming heat on the exterior surface. The outgoing heat is zero on the interior surface. On the exterior surface, the outgoing heat is higher than the incoming one due to cold nights. The balance over the wall leads to similar values in gain and loss.

For the climate of Toulouse, similar results are observed. However, due to both higher incoming heat and lower outgoing heat on the exterior surface, the heat balance of the wall is slightly positive, and the wall heats up slightly.

For the climates of Kairouan and Djerba, on the interior surface, light incoming and outgoing heat are observed. This leads to limited heat gain trough the wall under summer climate, without solar gain. On the exterior surface, incoming heat is higher than outgoing. Finally, the heat balance of the wall is positive and induces heat storage.

The numerical results are in the same trend as the experimental ones: (i) they show the same ranking of the cities according to the incoming and outgoing heat on each surface and in total, (ii) the highest heat between incoming and outgoing is the same for a given city on each surface and in total. On the interior surface, the numerical results are close to the experimental ones for the climates of Rennes and Toulouse, they slightly underestimate the experimental ones for Kairouan and Djerba. On the exterior surface, the numerical results always overestimate the experimental ones, by 14% for Kairouan and up to 59% for Rennes. As a result, both the total incoming and outgoing heats are numerically overestimated. However, the numerical heat balances between the incoming and outgoing heats are in the same range as the experimental ones, with values ranging from 3 Wh/m^2^ for Rennes to 30 Wh/m^2^ for Kairouan, linking with climate conditions. This positive heat balance explains the small temperature beam increase observed on the kinetics even for the last cycle.

**Figure 23:**
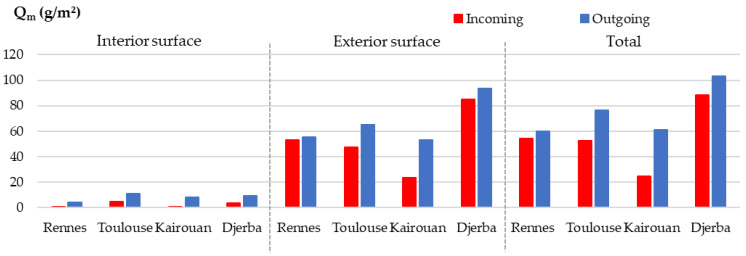
Numerical incoming and outgoing moisture during the last cycle on each surface of the wall and total of both surfaces.

Figure 23 gives the incoming and outgoing moisture calculated by integrating numerical fluxes versus time on each surface of the wall and the total of both surfaces.

On the interior surface of the wall, the incoming and outgoing moisture are very low for all the cities, with incoming values ranging from 1 to 5 g/m^2^ during the last cycle and outgoing values ranging from 4 to 11 g/m^2^. This is linked to the regulation of the indoor ambience at constant temperature and relative humidity and to the fact that the moisture flux coming from the outdoor side does not reach the first interior third of the wall thickness.

On the exterior surface, the incoming and outgoing moisture are much more important during the last cycle than the ones on the interior surface. Kairouan exhibits the lowest incoming moisture (24 g/m^2^), Rennes and Toulouse show intermediate values (about 50 g/m^2^) and Djerba the highest (85 g/m^2^). The outcoming moisture is similar for Rennes, Toulouse and Kairouan (53 to 65 g/m^2^) and much higher for Djerba (93 g/m^2^).

Finally, the moisture balance is negative for all the cities, the lowest for Rennes, and the highest for Kairouan. In accordance with the results on the vapor pressure kinetics, the wall is drying.

## 4. Conclusions

This paper presents the experimental and numerical study of the hygrothermal response of a bio-based wall under French and Tunisian typical summer climates, without considering solar gains (north walls, cloudy days, sunscreen effect).

The wall is made of a washing fines hemp composite layer with a hemp-lime render on the outdoor side and a skincoat clay plaster on the indoor side.

The experimental study, performed with a bi-climatic device, includes a stabilization phase under constant temperature and vapor pressure gradients and a dynamic phase with daily cyclic variations. The numerical study is performed with WUFI Pro 6.5 software. It investigates the same solicitations phases, completed with a study under constant temperature and vapor pressure gradient up to steady state.

The conductive thermal resistances of the wall calculated from the experimental and the numerical profiles and fluxes are in high agreement with the theoretical value (2.59 m^2^/(K·W)) at the end of the stabilization phase. The hygric internal resistances of the wall calculated from numerical results show discrepancy with the theoretical value at the end of the stabilization phase. However, when the simulation is extended to a steady state, the theoretical value is met (7.535·10^9^ m^2^·s·Pa/kg). The simulation of the stabilization phase shows that the evolution of the numerical profiles versus time are in good agreement for temperature but delayed for vapor pressure. This may be due to underestimation of the vapor diffusion, which is calculated from the vapor permeability and the main adsorption curve.

For daily cycles, two thirds of the thickness of the wall on the exterior side are active, both for heat and moisture phenomena. At mid-thickness, the shift of the signal is about 3 h both for temperature and for vapor pressure. The theoretical shift of the wall is about 12 h, what is interesting in terms of smoothing heat gains and losses. The temperature amplitude is damped over the thickness of the wall, with a damping factor of 0.2 between the exterior surface and the mid-thickness. On the interior surface, there is nearly no temperature variation. Sorption–desorption phenomena are highlighted in the exterior part of the wall, they are magnified with temperature variation. Finally, thanks to the heat and hygric inertias of the wall, there is a limited impact of the outdoor temperature and humidity variations on the indoor ambient conditions, through the wall, when there is no solar radiation on the wall.

The numerical results obtained from WUFI Pro software are in good agreement for temperature profile and heat balance but slightly overestimate the vapor pressure variations in the wall and underestimate the moisture diffusion.

Finally, as expected from the material properties, this wall is relevant for the studied summer conditions. To fully assess its relevance, complementary studies could take into account solar radiation, other seasons and locations.

## Figures and Tables

**Table 1:** Experimental sensors’ positions.

Sensor	1	2	3	4	5	6	7	8	9	10
**Position × (cm)**	Int	0	0.3	5.3	11.3	17.1	23.7	28.3	29.5	Ext

**Table 2:** Numerical monitors’ position.

Monitor	a	b	c	d	e	f	g	h	i	j
**Position × (cm)**	Int	0	0.04	4.46	10.71	16.99	23.10	27.99	29.5	Ext

**Table 3:** Physical properties at dry state of materials.

Material	ρ_0_ (kg/m^3^)	ε_0_	μ_0_	λ_0_ (W/(m·K))	C_p0_ (J/(kg·K))	W_80_ (kg/m^3^)	w_m_ (g/g)	C_1_	C_2_
LHR [34]	785	0.631	13	0.28	1006 *	36.08	0.02	3.66	0.89
WFH [35]	448	0.76	4.3	0.11	1250 *	23.37	0.02	3.66	0.89
SCP [40]	1514	0.42	11.3	0.65	850	18.8			

Where ρ_0_ is the dry density (kg/m^3^), ɛ_0_ is the total porosity (−), µ_0_ is the dry water vapor resistance factor (−), λ_0_ is the dry thermal conductivity (W/(m·K)), C_p0_ is the dry thermal capacity (J/(kg·K)), W_80_ is the volume water content at 80% (kg/m^3^), w_m_ is the monomolecular water content (g/g), C_1_ and C_2_ are the fitting parameters of the GAB model. * estimated values.

**Table 4:** Experimental heat fluxes and R_c_ values at the end of the stabilization phase.

	T_2_exp_ (°C)	T_9_exp_ (°C)	ϕ_h int_exp_ (W/m^2^)	ϕ_hext_exp_ (W/m^2^)	R_c, int_exp_ (m^2^·K/W)	R_c, ext_exp_ (m^2^·K/W)	R_c, exp_ (m^2^·K/W)
Rennes	22.45	14.34	3.09	3.49	2.62	2.32	2.470
Toulouse	22.53	15.82	2.62	2.74	2.564	2.554	2.509
Kairouan	25.46	20.92	1.96	1.74	2.319	2.610	2.464
Djerba	25.58	22.49	1.15	1.14	2.694	2.704	2.698

**Table 5:** Numerical heat fluxes and R_c_ values at the end of the stabilization phase.

	T_2_num_ (°C)	T_9_num_ (°C)	ϕ_h int_num_ (W/m^2^)	ϕ_h ext_num_ (W/m^2^)	R_c, int_num_ (m^2^·K/W)	R_c, ext_num_ (m^2^·K/W)	R_c, num_ (m^2^·K/W)
Rennes	22.52	14.24	3.08	3.67	2.685	2.255	2.470
Toulouse	22.73	15.85	2.54	2.83	2.706	2.435	2.571
Kairouan	25.58	20.86	2.03	1.64	2.331	2.888	2.610
Djerba	25.75	22.20	1.54	1.78	2.304	2.000	2.152

**Table 6:** Numerical heat fluxes and R_c_ values after 180 days under constant temperature and vapor pressure gradients.

	T_2_num_ (°C)	T_9_num_ (°C)	ϕ_h int_num_ (W/m^2^)	ϕ_h ext_num_ (W/m^2^)	R_c, int_num_ (m^2^·K/W)	R_c, ext_num_ (m^2^·K/W)	R_c, num_ (m^2^·K/W)
Rennes	22.51	14.23	3.17	3.17	2.609	2.609	2.609
Toulouse	22.70	15.82	2.64	2.64	2.609	2.609	2.609
Kairouan	25.61	20.86	1.82	1.82	2.609	2.609	2.609
Djerba	25.74	22.19	1.36	1.36	2.608	2.608	2.608

**Table 7:** Numerical moisture fluxes and R_h_ values at the end of the stabilization phase.

	Pv_2_num_ (Pa)	Pv_9_num_ (Pa)	ϕ_m int_num_ (kg/ (m^2^·s))	ϕ_m ext_num_ (kg/ (m^2^·s))	R_h, int_num_ (m^2^·s·Pa/kg)	R_h, ext_num_ (m^2^·s·Pa/kg)	R_h_num_ (m^2^·s·Pa/kg)
Rennes	1477	1309	8.29·10^−8^	−1.93·10^−7^	-	-	-
Toulouse	1418	1397	8.13·10^−9^	−2.17·10^−8^	-	-	-
Kairouan	1841	1453	−2.00·10^−8^	1.20·10^−7^	-	-	-
Djerba	1846	2228	−2.35·10^−8^	−2.13·10^−7^	-	-	-

**Table 8:** Numerical moisture fluxes and R_h_ values after 300 days of stabilization.

	Pv_2_num_ (Pa)	Pv_9_num_ (Pa)	ϕ_m int_num_ (kg/ (m^2^·s))	ϕ_m ext_num_ (kg/ (m^2^·s))	R_h, int_num_ (m^2^·s·Pa/kg)	R_h, ext_num_ (m^2^·s·Pa/kg)	R_h_num_ (m^2^·s·Pa/kg)
Rennes	1483	1305	2.78·10^−8^	2.62·10^−8^	6.40·10^9^	6.78·10^9^	6.59·10^9^
Toulouse	1410	1394	2.23·10^−9^	2.26·10^−9^	7.14·10^9^	7.05·10^9^	7.10·10^9^
Kairouan	1833	1451	5.84·10^−8^	5.84·10^−8^	6.54·10^9^	6.54·10^9^	6.54·10^9^
Djerba	1845	2243	−5.67·10^−8^	−5.73·10^−8^	7.01·10^9^	6.95·10^9^	6.98·10^9^

## Data Availability

For the data supporting, please contact the corresponding author.
